# Transfer Learning Allows Accurate RBP Target Site Prediction with Limited Sample Sizes

**DOI:** 10.3390/biology12101276

**Published:** 2023-09-25

**Authors:** Ondřej Vaculík, Eliška Chalupová, Katarína Grešová, Tomáš Majtner, Panagiotis Alexiou

**Affiliations:** 1 Central European Institute of Technology (CEITEC), Masaryk University, 625 00 Brno, Czech Republic; 2 Faculty of Science, National Centre for Biomolecular Research, Masaryk University, 625 00 Brno, Czech Republic; 3 Department of Molecular Sociology, Max Planck Institute of Biophysics, 60439 Frankfurt am Main, Germany; 4 Department of Applied Biomedical Science, Faculty of Health Sciences, University of Malta, MSD 2080 Msida, Malta; 5 Centre for Molecular Medicine & Biobanking, University of Malta, MSD 2080 Msida, Malta

**Keywords:** RNA-binding protein, CLIP-seq, deep learning, transfer learning, interpretation

## Abstract

**Simple Summary:**

RNA-binding proteins play crucial roles in essential biological processes, and disruptions in their functionality can lead to various diseases, including cancer. Despite the significant progress that computational deep learning methods have made in identifying their binding sites, obtaining high-quality data in sufficient amounts remains a major challenge, impeding development of accurate predictive models for many proteins. In this work, we present a novel approach to address the limited availability of training samples by leveraging transfer learning for predicting RBP binding sites. Using three input features and a sophisticated network architecture, we demonstrate the substantial advantages of employing transfer learning in a reusable and interpretable manner, as showcased on two prominent benchmark datasets for RNA-binding proteins.

**Abstract:**

RNA-binding proteins are vital regulators in numerous biological processes. Their disfunction can result in diverse diseases, such as cancer or neurodegenerative disorders, making the prediction of their binding sites of high importance. Deep learning (DL) has brought about a revolution in various biological domains, including the field of protein–RNA interactions. Nonetheless, several challenges persist, such as the limited availability of experimentally validated binding sites to train well-performing DL models for the majority of proteins. Here, we present a novel training approach based on transfer learning (TL) to address the issue of limited data. Employing a sophisticated and interpretable architecture, we compare the performance of our method trained using two distinct approaches: training from scratch (SCR) and utilizing TL. Additionally, we benchmark our results against the current state-of-the-art methods. Furthermore, we tackle the challenges associated with selecting appropriate input features and determining optimal interval sizes. Our results show that TL enhances model performance, particularly in datasets with minimal training data, where satisfactory results can be achieved with just a few hundred RNA binding sites. Moreover, we demonstrate that integrating both sequence and evolutionary conservation information leads to superior performance. Additionally, we showcase how incorporating an attention layer into the model facilitates the interpretation of predictions within a biologically relevant context.

## 1. Introduction

RNA-binding proteins (RBPs) are essential in a wide range of biological processes during the RNA lifecycle, from transcription, through splicing and transport, to translation [1]. Their importance is evident as disrupted function or expression and indicated in diverse diseases, such as cancer or neurodegenerative disorders [2]. Characterizing RBP binding sites across the transcriptome helps uncover their regulatory roles and functionality, facilitating, for example, a greater understanding of cellular physiology and disease pathology [3]. To date, more than 2000 human RBPs are known [4,5]. While there are a few thoroughly studied and well-described proteins, we still lack a deeper understanding of the remaining vast majority, including thousands of newly recognized RBPs with unknown binding modes [6].

One of the currently most widely used experimental approaches for the localization of RBP binding sites is UV cross-linking followed by immunoprecipitation and high-throughput sequencing (CLIP-Seq) [7]. Multiple modifications of the CLIP-Seq protocol have already been developed, continuously improving various aspects, such as the precision of the binding site localization [8]. So far, CLIP-Seq has been successfully applied in many studies, and has helped, for example, to characterize the binding profiles of several RBPs involved in neurologic disorders and cancer [9].

Despite continuing progress, experimental protocols remain costly and time-consuming and suffer from issues like low RNA purification levels or inefficient crosslinking [10,11], which makes them unsuitable for the complete transcriptome-wide binding site discovery across the broad range of RBPs. However, the acquired experimental data can be sufficient as the basis for computational methods to fill in the missing binding sites not detected by the experiment.

The prediction of RBP binding sites has a well-established history, starting with the utilization of sequence-motif discovery tools [12,13,14]. With this approach, motifs enriched in the experimental data are identified by statistical modeling and then searched for the target RNA sequence using various filtering and selection criteria [12,15]. Other methods utilize various machine learning (ML) algorithms, such as Support Vector Machines [16,17] or nonnegative matrix factorization [18], to classify potential binding sites. Although the ML tools are more robust than their predecessors, they require deep domain knowledge and hand-crafted input features, introducing a human bias into the process.

On the other hand, deep learning (DL), a subset of ML, has gained tremendous popularity in recent years due to its ability to learn such features from raw data automatically without any feature engineering, given sufficient training data available. This has enabled DL to be successfully applied to various biological problems, including protein–RNA binding [19]. The first DL-based method, DeepBind, used a CNN to predict RBP binding sites from RNA sequences [20] and was followed by a number of other tools that used various architectures and input features, such as iDeepS [21] and DeepRiPe [22]. PrismNet was the first method to use in vivo RNA secondary structure information and implemented an attention layer in its hybrid architecture [23]. Although the current state-of-the-art DL tools already achieve remarkable results on benchmark collections [24,25], their performance is often lower on proteins with smaller datasets (Supplementary Table S1). Considering the proportion of poorly characterized RBPs, there are many proteins with an insufficient number of experimentally identified binding sites to train a well-performing DL model.

An optimization process called transfer learning (TL) has been developed to overcome the dataset size limitations and save resources when training new models [26]. The principle lies in reusing the information extracted from a previously learned task as starting general knowledge when learning a new task. The technique has been shown to reduce the required amount of training data while improving the overall model performance for diverse applications. TL has been widely acclaimed for its significant contributions across various domains, including genomics research. For instance, it has demonstrated successful outcomes in predicting genomic features [27], chromatin interactions [28], or transcription-factor binding sites [29,30].

In this work, we present a novel approach for predicting RBP binding sites based on transfer learning to tackle the problem of a small number of training samples. Our approach is based on our prior research [31], where we used three different inputs—RNA sequence, evolutionary conservation, and predicted secondary structure—in order to train a three-branch DL model. The branches were built using either an attention-based hybrid architecture or residual networks [32]. The attention hybrid architecture combines the strengths of Convolutional Neural Networks (CNNs) and Recurrent Neural Networks (RNNs) with an attention layer, providing a complex model that learns the binding rules from the data both when trained from scratch (SCR) and using TL. Our results on two widely used benchmark RBP data collections show that our models perform competitively compared to existing tools. We highlight the advantages of transfer learning in scenarios with a low amount of training data. Specifically, we evaluate the predictive performance of our method on datasets with limited data, as well as its ability to be fine-tuned on previously unseen data. This study provides a new direction for researchers working on model development for poorly characterized proteins. The code and data are available at https://github.com/VaculikOndrej/TransferLearningRBP (accessed on 14 August 2023).

## 2. Materials and Methods

### 2.1. Datasets

To investigate the benefits of TL for the RBP binding site prediction, we applied the approach to the two most widely used CLIP-seq benchmark collections of RBP datasets—RBP-24 [17] and RBP-31 [18]. The original fasta files for RBP-31 can be downloaded from the iONMF repository at https://github.com/mstrazar/ionmf (accessed on 14 August 2023), and for RBP-24 from the GraphProt repository at http://www.bioinf.uni-freiburg.de/Software/GraphProt/ (accessed on 14 August 2023). Both datasets differ from each other in several important properties. The RBP-24 dataset contains RNA sequences with variable lengths ranging from 150 to 375 nucleotides (nt), while the RBP-31 dataset contains fixed-length RNA sequences of 101 nt. The nonbinding negative sites were derived differently in each dataset: in RBP-24 by shuffling the coordinates of binding sites within all genes with at least one binding site, and in RBP-31 by extracting positions from any of the 31 experiments that were not identified as interacting. Moreover, there is a big difference in the ratio of positives to negatives. RBP-24 contains balanced datasets, whereas RBP-31 comprises datasets with a more unbalanced ratio of 1:4 of positives to negatives. As a final significant difference, the number of RNA binding sites (samples) in each dataset in RBP-24 ranges from only a few hundred samples to over one hundred thousand samples for the largest dataset. On the other hand, all datasets in RBP-31 contain the same number of samples, which is also significantly low, and, in combination with the unbalanced ratio, considerably challenging for classification.

All these differences result in the need for different base models (BS), as it would not be possible to efficiently fine-tune a BS trained on one of those datasets on the individual datasets of the other one. Also, combining both datasets into one ‘baseline’ dataset was not possible, especially due to a different ratio of positives to negatives and the much smaller total size of the RBP-31 dataset.

### 2.2. Input Features and Encoding

We based our method on three different input features: RNA sequence, predicted RNA secondary structure, and evolutionary conservation. In order to obtain additional features of the RNA sequence, we first extracted genomic coordinates from the sequence headers of the original fasta files. Subsequently, the obtained coordinates were preprocessed the same way as in [32], resulting in 150 nt long RNA sequences centered at the initial coordinates. The length of 150 nucleotides has been previously used in several RBP target site predictors [31,33] and it was shown to be an optimal choice for RNA secondary structure prediction [34]. For the sake of additional analysis, we have created intervals of varying lengths, ranging from 100 nt to 300 nt, with 50 nt steps, for all datasets in both benchmarks.

For the evolutionary conservation feature, the genomic coordinates were mapped to the PhyloP100 conservation scores [35] from the PHAST package [36] obtained from the UCSC file storage (http://hgdownload.cse.ucsc.edu/goldenpath/hg19/phyloP100way/) (accessed on 14 August 2023). According to the original dataset specification [17], the PTBv1 dataset from RBP-24 was mapped to the older hg18 genome reference and the corresponding PhyloP files with the human genome and other 43 vertebrate genomes aligned (https://hgdownload.soe.ucsc.edu/goldenPath/hg18/phyloP44way/) (accessed on 14 August 2023).

To pre-train the base model, we generated two baseline datasets, one from each benchmark dataset, where all the training positive and negative samples were merged. We removed negative samples that overlapped with a binding site of any included protein and a corresponding number of positive samples to preserve the original positive-to-negative ratio. For the training itself, the baseline datasets were further split into training and validation sets in a ratio of 9:1. Evaluation sets of individual proteins were kept unchanged. To assess the effect of transfer learning on data not presented to the base model during the pre-training phase, one selected protein (PTBv1) was excluded from the baseline dataset.

RNA sequences (S) were encoded using a byte-pair encoding (BPE) [37] tokenizer trained on the whole human hg19 transcriptome. It was downloaded from the UCSC Table Browser (https://genome.ucsc.edu/cgi-bin/hgTables) (accessed on 14 August 2023) using track GENCODE V38lift37 with wgEncodeGencodeBasicV38lift37 table settings. BPE is a sub-word segmentation algorithm commonly used in Neural Machine Translation, for example, in the GPT2 [38] and RoBERTa [39] language models [40]. After the number of tokens that the tokenizer should search for is defined, the algorithm begins by searching for the smallest tokens in the training corpus—in our case, the individual nucleotides. Afterward, additional tokens are created by merging the most frequent pairs of tokens until the defined number of words (k) is reached [41,42].

In order to tokenize the sequence using the vocabulary, the tokenizer initializes an empty list to store the tokens and iterates through the sequence. It searches for the longest matching token from the vocabulary that can be identified for the current substring. Once it is found, the matched token is appended to the list of tokens and is removed from the substring. The process is repeated until the entire sequence is tokenized into a sequence of tokens, each of which corresponds to a token from the vocabulary. This process results in no unknown words in the tokenized sequences.

As the suitable number of tokens depends on the complexity of the text, we decided to develop three separate tokenizers, with k = {16, 32, 64}. Numbers are based on the number of di-nucleotides and codons plus the value of 32 as a natural intermediate step. We also briefly examined the higher values. However, our results showed no improvement, only a longer preprocessing time when preparing the tokenizer.

The predicted secondary structure (SS) was computed using the ViennaRNA2 package [43] in the simple dot-brackets format. Given a sequence of RNA secondary structure symbols, s = (r1, r2, …, rn) with n nucleotides, it was then encoded into a one-hot matrix M with a size n × 3 as below:

(1) $$M_{i,j}=\{1\;\mathrm{if}\;ri\;=\;j\text{th}\;\text{base}\;\text{in}\;[.\;(\;)]\;\text{else}\;0\},$$

where *i* is the index of the base-wise structure along the sequence and *j* is the index for one of the symbols {., (, )}.

Evolutionary conservation (EC) scores were mapped to the coordinates based on the input bed files, resulting in arrays of floating-point numbers. Each position in the array represents the corresponding score of a given nucleotide in the genome.

### 2.3. Deep Learning Architecture

**Figure 1:**
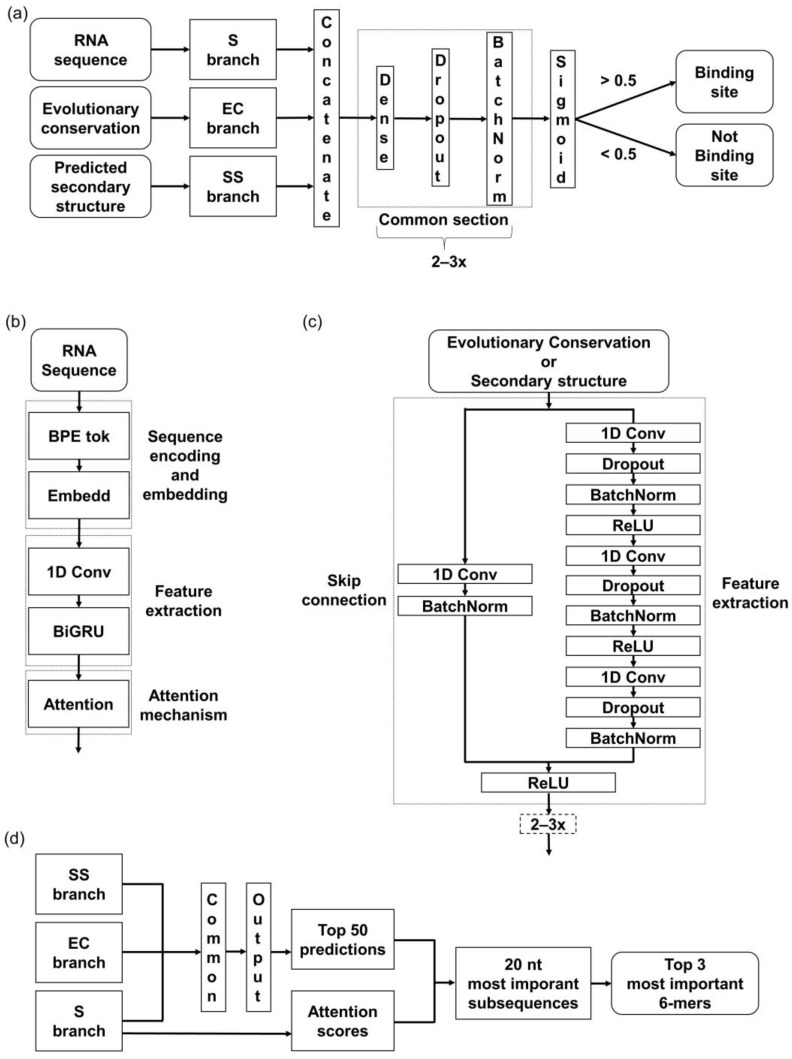
Model architecture overview. Individual branches and all the abbreviations are described in detail in Section 2.3. (**a**) Brief scheme of the used model architecture with a detailed look at the Common section of the model that processes the concatenated outcomes from individual branches to provide a final prediction. (**b**) A detailed look at the Sequence branch. (**c**) A detailed look at the Evolutionary Conservation and Secondary structure branches. (**d**) Overview of the interpretation method used in this paper. The methodology behind this is described in Section 2.7.

The developed model architecture consists of three branches, corresponding to the above-mentioned input features—RNA sequence (S), predicted secondary structure (SS), and evolutionary conservation (EC) (Figure 1a). In the S branch, our method combines a hybrid CNN-RNN architecture with an attention mechanism (Figure 1b). First, S is tokenized into a sequence of tokens. Afterward, the embedding layer creates a denser representation of tokens while maintaining mutual relationships. The 1D-convolutional layer is applied to extract the local contextual information from the sequences, followed by a bi-directional gated recurrent unit (BiGRU) layer to extract the long-range global features. Lastly, the attention layer is applied to boost the contribution of the critical features by assessing and enhancing their importance.

The other two branches, SS and EC, are based on the CNN architecture, specifically the Residual Network in the form of so-called ResNet blocks (Figure 1c). ResNet blocks consist of a sequence of convolutional layers and a skip connection block (or the so-called residual block). The main advantage of using ResNet instead of the common 1D-convolutional layers is an improvement of the gradient flow throughout the network [43]. The skip connections enable a block to be skipped if any of its layers negatively impact the model’s performance during the training. Specifically, the skip connection allows the output from an earlier layer in the block to be directly passed to a later layer, bypassing intermediate layers that may cause problems with overfitting. This way, a deeper architecture can be trained without losing performance.

Outputs from all three branches are concatenated and processed through the last section, composed of fully connected dense layers. The sigmoid function at the last layer determines whether the sequence does or does not contain a binding site (Figure 1a).

### 2.4. Evaluation Metrics

In order to ensure a standardized performance measurement, we used the area under the receiver operating characteristic curve (AUC) as the evaluation metric. The AUC metric was applied by all the previous RBP binding site prediction tools cited throughout the article, allowing for a straightforward performance comparison across the methods. The AUC is drawn between the false positive rate and the true positive rate. The mean AUC measures the ability of a model to distinguish between the defined classes and is calculated as follows:

(2) $$\mathrm{AUC}=\frac{\left(TP+FP\right)-\;\frac{TP+FN\times (TP+FN+1)}{2}}{TP+FP+TN+FN}$$

where *TP* is a shortcut for True Positives, *FP* for False Positives, *TN* for True Negatives and *FN* for False Negatives.

### 2.5. Base Model Pretraining and Transfer Learning

We trained 112 models with the same architecture (described in the section Deep Learning Architecture) and hyperparameters (described in the section Hyperparameter Optimization of a Base Model) but using different datasets and training approaches. Trained models can be divided into three groups: base models (BS), models trained from scratch (SCR), and models trained using transfer learning (TL).

First of all, we trained two BS models, one for each baseline dataset. These models are designed to extract common features from the RBP binding sites and distinguish them from genomic intervals without any binding site. Trained BS models were used as a starting point for fine-tuning 55 TL models—one for each dataset in RBP-31 and RBP-24. Additionally, we trained 55 SCR models on the same data as TL models, but we used randomly initialized models as a starting point. All the models were evaluated using the left-out evaluation sets.

Our models were trained and optimized on the Ubuntu 20.04 PC with the following hardware parameters—AMD Ryzen Threadripper 2920X 12-Core CPU, GeForce RTX 2080 Ti 11GB GPU, and 128GB RAM.

### 2.6. Hyperparameter Optimization of a Base Model

To select a suitable combination of hyperparameters for the BS models, we developed a pipeline consisting of several subsequent stages. They are defined with respect to the individual parts of the network architecture in order to minimize the required processing time. First, the parameters for the S branch are optimized, followed by the tokenizer size optimization in the second stage. In the third stage, the hyperparameters for the EC and SS branches are optimized together, as these branches share the same architecture. Lastly, the common section of the model, composed of fully connected layers, is optimized. For every stage, the optimal hyperparameter values were selected from a search space based on the average AUC score obtained from a 10-fold cross-validation (CV) within the training baseline dataset, and the previously optimized parameters were taken as fixed in each following stage. The hyperparameter optimization was performed separately for each dataset due to different sizes and positive-to-negative ratios. To speed up the optimization process for the significantly larger RBP-24, we downsampled it to 1/4 of its original size while preserving the original positive-to-negative ratio. Individual steps and hyperparameter values are shown in Table 1.

We compared our pipeline’s time efficiency and performance with that of a commonly used hyperparameter optimization algorithm, Random Search (RS), on the RBP-31 dataset. The RBP-31 dataset was chosen for its smaller size. As there are 27 possible combinations of hyperparameters in our pipeline due to the “staged” approach, we set up the RS for 27 trials. We preserved the number of executions per trial and the search space the same as in our pipeline and ran the optimization process 5 times. Finally, we compared the average running times of the two approaches.

### 2.7. Attention Score as Proof of Learning RBP Specifics

To validate that our models focused on the relevant features, we utilized the attention mechanism incorporated in the network architecture. We made predictions on all the evaluation sets and separated the top 50 RNA sequences predicted as bound with the highest probability. We extracted 20 nt long regions with the highest average attention score from these. Within those regions, we searched for the most frequent 6-mers (Figure 1d) and compared the obtained *k*-mers with the motifs from the literature.

## 3. Results

### 3.1. Improved Time Efficiency with Our Proposed Hyperparameter Optimization Pipeline

First, we evaluated the time efficiency of our hyperparameter optimization algorithm in comparison to the Random Search optimization. As shown in Table 2, our pipeline substantially improved the time efficiency of the optimization process while keeping the performance of the optimized model. Our results also showed a minor but significant improvement in the average performance of the models derived from our method compared with RS optimization (*p*-value: 1.3 × 10^−2^). Since our proposed pipeline is approximately 1.4 times faster on average and incorporates the tokenizer size into the search space, which is not possible using RS optimization, we decided to use it in all our experiments.

### 3.2. Fine-Tuned Models Outperform Models Trained from Scratch for RBP Binding Site Prediction

**Figure 2:**
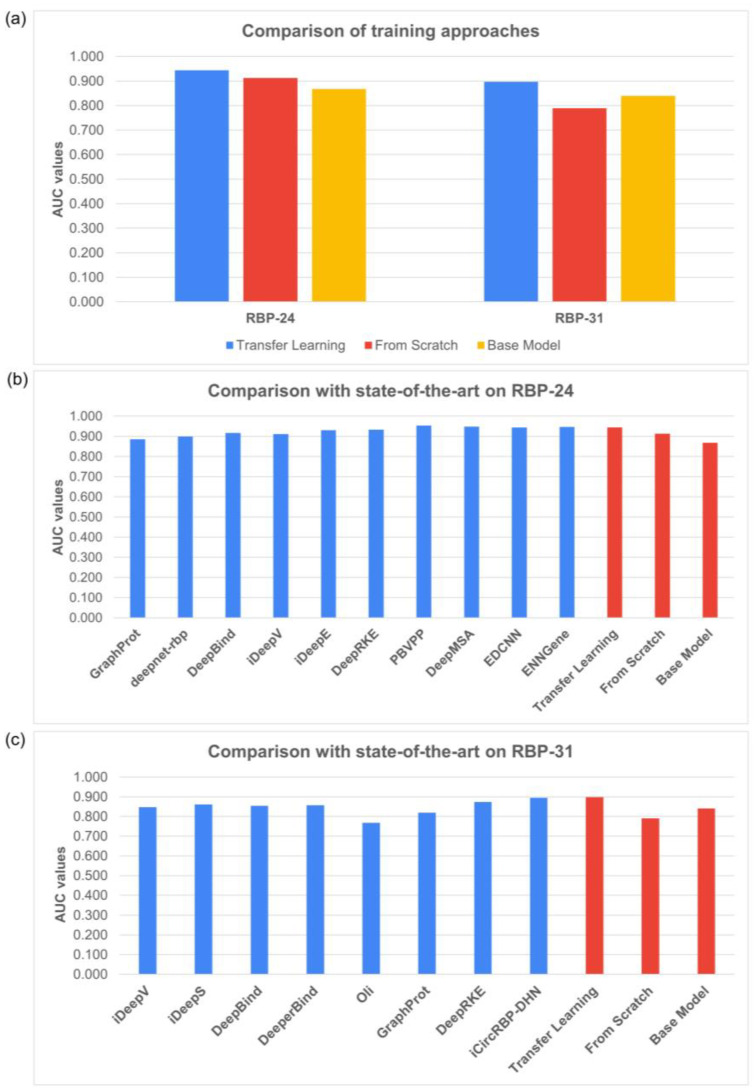
Comparison of average AUC scores on RBP-24 and RBP-31 datasets. (**a**) Comparison of our method trained using different approaches. (**b**) Performance comparison of our methods to other published methods on RBP-24 dataset. (**c**) Performance comparison of our methods to other published methods on RBP-31 dataset. The base model’s performance differs widely in individual benchmark datasets (**b**,**c**) and the possible solution is discussed in the Discussion Section 4. The presented AUC scores for individual methods were obtained from [15,17,20,21,24,31,33,44,45,46,47,48,49].

To examine the effect of transfer learning versus training models from scratch, we applied both approaches to the RBP-24 and RBP-31 benchmarks. As shown in Figure 2a, the results reveal that transfer learning improved prediction performance on both datasets. The average AUC scores achieved by scratch models were significantly lower on both datasets, particularly on the more challenging RBP-31 dataset. To facilitate comparison, we also evaluated BS models on individual proteins without the fine-tuning step.

To emphasize the strength of the proposed TL method, we compared our SCR and TL results to other published methods on RBP-24 and RBP-31 (Figure 2b,c). Our TL models matched the performance of the current state-of-the-art methods and even surpassed them on the RBP-31 dataset. SCR models ranked among the poorer-performing models, especially on the RBP-31 dataset. More detailed results may be found as figures in the Supplementary Materials (Supplementary Figures S1 and S2).

### 3.3. Interval Length Does Not Have a High Impact on the Model Prediction Ability

We took advantage of the fact we work with interval inputs instead of the typically used fasta files. Adjusting the interval sizes from 100 nt to 300 nt with 50 nt increments, we have examined the impact of removing or adding information to the model. A BS model for each interval size was trained and fine-tuned on the individual datasets.

**Figure 3:**
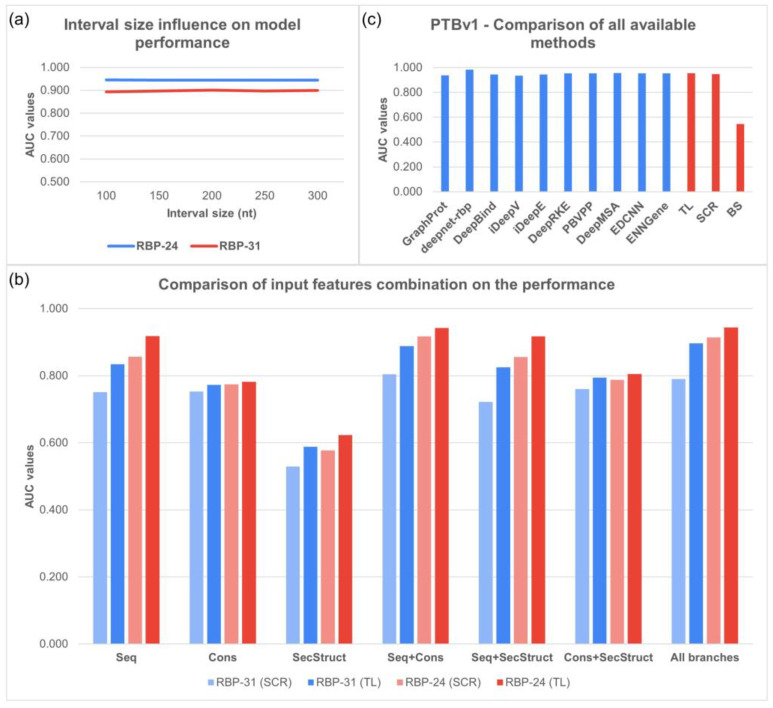
(**a**) Impact of interval length on models’ average performance. We observed no change in performance when the interval size was shortened or extended. (**b**) Performance comparison of both training approaches using various combination of input features. (**c**) Performance comparison of our methods to other published methods on the left-out PTB dataset from RBP-24. The very low BS model performance on the PTBv1 dataset is caused by its limited predictive ability on unseen proteins and highlights the importance of the fine-tuning step. The presented AUC scores for individual methods in c) were obtained from [17,20,24,31,33,44,45,47,48,49].

As shown in Figure 3a, the scores for RBP-24 indicate that most proteins performed better on shorter genomic intervals, with a slight decrease in performance on longer intervals. However, there are exceptions, such as CLIPSEQ_ELAVL1, which demonstrated consistently high prediction scores across all interval lengths. The average AUC score for RBP-24 is 0.944.

In RBP-31, the performance for most proteins varied slightly with the length of the RNA interval. While some proteins demonstrated better performance with shorter intervals, others showed the opposite trend. The AUC score for RBP-31 is 0.897.

### 3.4. Incorporating Evolutionary Conservation Significantly Improves the Performance

We further examined the contribution of particular input features and their combinations to the RBP binding site prediction performance. We analyzed the SCR and TL approaches separately to identify the differences. As S is the most common input feature and many methods use it as the only input, we considered the scores obtained on the RNA sequences as a baseline. Figure 3b contains all the average AUC scores obtained for each dataset.

Starting with SCR models, S provided the best overall results from the single-input-feature models, followed by EC. The results obtained from models trained only on SS were close to random predictions. Adding EC to S significantly improved the average AUC performance on both benchmarks, representing an improvement of 5.3% and 6.0%, with a noticeable increase (>1%) in 19 proteins in RBP-31 and 14 proteins in RBP-24. On the other hand, adding SS to S did not improve the predictions. With SS added to the EC, the performance improved by 0.7% in RBP-31 and 1.4% in RBP-24 in comparison to simple EC models. Combining all three inputs outperformed the S models on both benchmarks. However, the scores remained below the S+EC combination by 1.4% in RBP-31 and 0.3% in RBP-24.

Using the TL approach, the S models performed even better compared with other input features, exceeding the EC models by 6.1% and 13.6%. The average AUCs in SS models were again close to random predictions. A combination of S and EC significantly improved the scores on both benchmarks, while adding SS to S brought a minor performance drop in RBP-31 and an almost identical average AUC for RBP-24, which means a similar situation to the SCR models. Adding SS information to EC gave us an increase of 2.1% in RBP-31 and 2.3% in RBP-24 compared with EC-only models. Nevertheless, the performance of such models remained far behind the S-only models. Interestingly, combining all three input features in the TL method showed the best overall scores for both datasets, reaching 89.7% in RBP-31 and 94.4% in RBP-24. Even though the increase was only by 1.0%, resp. 0.2%, when compared to the second-best combination of S+EC, both changes are statistically significant (Wilcoxon Signed-Rank test, *p*-value: 3.3 × 10^−4^ for RBP-31, resp. *p*-value: 2.8 × 10^−3^ for RBP-24).

### 3.5. Transfer Learning Enables Powerful Predictions on Limited Datasets

We left the PTBv1 data out of the baseline set and BS model pretraining (described in Methods Section 2, Datasets and input features) and used it to test the model’s ability to be fine-tuned on novel unseen data. For comparison, we have produced two additional models on the PTBv1 dataset, one trained from scratch and one using the transfer learning approach. Additionally, we compared both models with other published methods to put our results into a broader context.

Evaluating the BS model on the PTBv1 dataset revealed its limited predictive ability on unseen proteins, with prediction scores close to random. In contrast, the SCR and TL models significantly improved the predictive performance, surpassing the 94% AUC score. Notably, the TL model outperformed the SCR model by 0.6% AUC, achieving a highly competitive score of 95.3%. Reaching a high score comparable to other published models (Figure 3c) trained directly on the dataset underscores the potential of TL models in improving the accuracy and efficiency of RBP binding site prediction.

**Figure 4:**
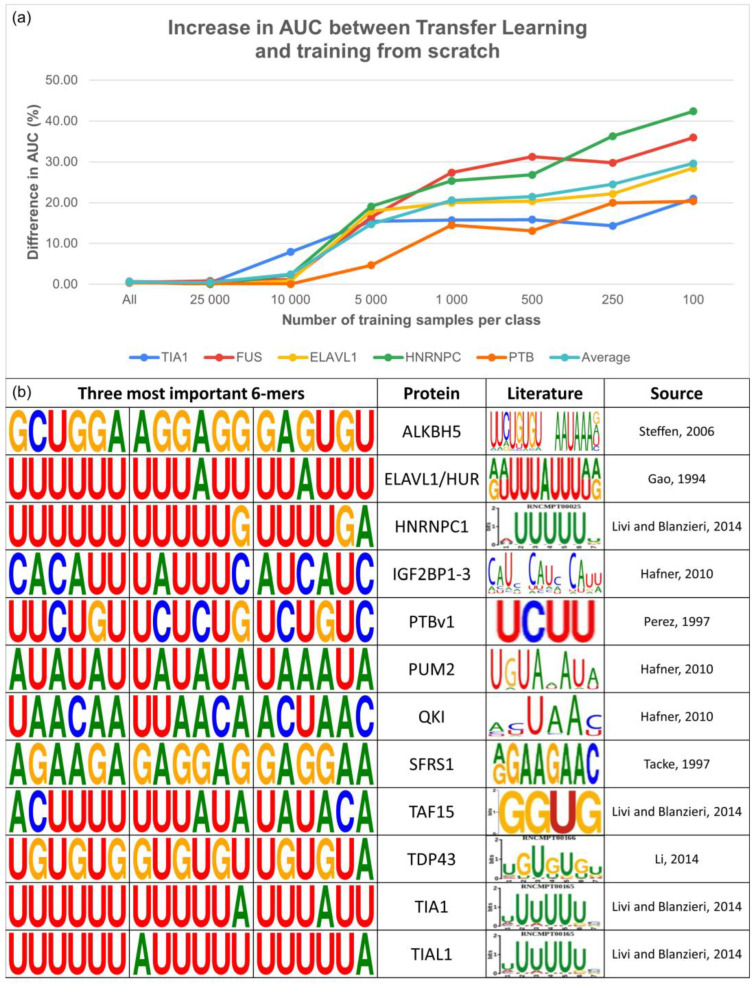
(**a**) Performance comparison of both training approaches on several datasets based on the amount of available training data. The performance was measured in the form of AUC and the results are expressed in percent. The results show an enormous performance increase when the TL approach is used on datasets with limited training data of less than 10 thousand samples (Paired Sample *t*-Test, *p*-value ranges: 6.40 × 10^−4^ to 2.35 × 10^−3^ for datasets containing 100–5000 samples). (**b**) Comparison of the RNA subsequences detected by our models as the most important for the binding site determination with the binding motifs derived from literature [15,50,51,52,53,54,55].

As the PTBv1 dataset is one of the largest in RBP-24, we further selected four additional proteins and used them along with the PTBv1 dataset to investigate how efficiently our method can learn from a limited number of samples. For this purpose, we chose PARCLIP_ELAVL1, ICLIP_TIAL1, ICLIP_HNRNPC, and PARCLIP_FUS, which range between 20 and 36 thousand samples per class. We subsampled all of them to the following sizes per class: 100, 250, 500, 1000, 5000, 10,000, and 25,000 (where possible), and the entire dataset. With each increase in the number of samples, we ensured that the subsequent datasets contained all the previous binding sites, with an adequate number of new ones. Afterward, we trained the SCR models on each subsampled dataset and fine-tuned the BS model as described in previous sections. SCR and TL models were trained ten times in total, with the results representing the average AUC values for every approach per dataset (Figure 4a).

The obtained results unanimously demonstrated the ability of our TL method to be fine-tuned on even a very limited number of samples, achieving substantial improvements in the AUC score compared with the respective SCR models. Specifically, we observed an average improvement of 29.6% for datasets containing only 100 samples per class, 24.5% for 250 samples per class, 21.5% for 500 samples per class, 20.6% for 1000 samples per class, and 14.7% for 5000 samples per class. However, as the dataset size increased to 10,000, 25,000, and further, the improvement in the AUC score diminished while, importantly, never falling below the performance of the SCR models.

These results pinpoint the potential of TL models to overcome one of the most common limitations in RBP binding site predictions. By allowing to effectively train well-performing models even for the proteins with very limited datasets, TL opens up a new window of opportunity for improvement in the field.

### 3.6. Attention Scores as Proof of Learning RBP Specifics

In addition to the competitive prediction performance, we verified that our TL models learned appropriate features from the data through analysis of the attention scores. Attention scores are produced by an attention layer, which allows the model to focus on specific parts of the input data during prediction. In our case, the scores represent tokens within the RNA sequence and are calculated based on the contribution of each token to the final prediction output. This allows the model to assign higher weights to the most important parts of the sequence, thereby providing valuable insights into the binding characteristics of investigated RBPs and how the model makes its predictions.

Figure 4b demonstrates that the models were capable of detecting relevant RNA subsequences that differed between individual proteins with known binding preferences. These extracted *k*-mers were consistent with motifs derived from the literature, indicating that our models captured biologically relevant features. Additional *k*-mers for the remaining proteins in both datasets can be found in the Supplementary Materials (Supplementary Figures S3 and S4).

## 4. Discussion

In the present study, we performed a comprehensive assessment of the transfer learning approach for RBP binding site prediction. TL is a methodology that overcomes the challenge of training complex neural networks in scenarios with a limited amount of data samples available, thereby mitigating issues of overfitting and improving model accuracy. Apart from remarkable results in many other fields [56], TL has been successfully applied in various genomics research areas, including predicting genomic features [27], chromatin interactions [28], or transcription factor binding sites [29,30].

To thoroughly explore multiple aspects of transfer learning, we designed a three-branched Deep Learning model, with each branch processing a different input feature—RNA sequence, evolutionary conservation, or predicted secondary structure. The model architecture integrates several elements previously used with success in RBP target site prediction, such as the hybrid CNN-RNN network [44], attention network [25,45,57,58], or residual networks [59].

We applied our approach to the two most widely used CLIP-seq RBP benchmark collections, RBP-24 and RBP-31, to investigate the potential contribution of TL in the current state-of-the-art prediction of RBP target sites. These datasets differ in various crucial aspects (described in the Methods Section 2 Datasets and input features), making it infeasible to train a baseline model on both datasets collectively or cross-fine-tune protein models from a base model trained on the other benchmark dataset. Consequently, we tested our approach on each dataset individually.

Hyperparameter tuning is a crucial step in DL model development, identifying the optimal combination of hyperparameters that maximizes the model’s performance. However, commonly used algorithms, such as Random Search or Grid Search, are known to be time- and resource-intensive processes. Furthermore, it was not feasible to use Random Search or Grid Search algorithms for tuning the tokenizer size, as selecting the tokenizer size is a part of the data pre-processing stage, wherein RNA sequences are encoded using a specific number of words before providing them to the model for hyperparameter tuning/training. To reduce the tuning time and include tokenizer size in the search space, we proposed a novel hyperparameter tuning pipeline. To evaluate the effectiveness of our pipeline, we conducted a comparative analysis with RS on the RBP-31 dataset due to its smaller size, enabling faster assessment. Using the pipeline, we were able to reduce the tuning time by 19% on average.

Using the hyperparameter combinations obtained through our novel pipeline, we pre-trained the BS models, which were then fine-tuned on individual proteins through TL, as well as trained models for the individual proteins from scratch. The average AUC values showed a significant difference between TL and SCR models, with the difference more pronounced in RBP-31, possibly due to an insufficient number of samples for individual proteins. Additionally, results on the low abundant proteins in RBP-24 indicated an influence of the ratio of positives to negatives, with the balanced positive-negative ratio possibly allowing for well-performing SCR models. We plan to further investigate the cause of these findings in our future work, including the possibility that the observed performance differences may be partially attributed to the differently generated negatives in the RBP-31 and RBP-24 datasets.

We further looked at the performance of the BS models directly. Even without the fine-tuning step, the RBP-31 BS model outperformed the SCR models, suggesting it could sufficiently capture the binding features of the encompassed proteins. In contrast, the RBP-24 BS performed worse than the corresponding SCR models. The high variability in the number of samples in RBP-24 skews the patterns learned by the BS model towards the more abundant proteins, such as ELAVL1/HUR protein, with its well-known affinity to U- and AU-rich RNA sequences [60], that holds approximately 29% of all positive samples in the dataset. AU-rich elements are important for RNA regulation, found to be broadly involved in RNA processing, transport, and translation, and are bound by many RBPs [61]. On the contrary, we observed the biggest performance drop for the SRSF1 protein between our BS and SCR models. The SRSF1 protein binding motif is more distinct from other proteins in the dataset, as it prefers a purine-rich octamer ‘AGAAGAAG’ [62], meaning its binding characteristics could have been overshadowed by other, more frequently occurring motifs. These results show that while BS models can learn major binding patterns across proteins, they are insufficient on their own for proteins with atypical binding patterns and confirm the necessity of the fine-tuning step.

Various input features have been used to predict RBP binding sites, with RNA sequence and predicted secondary structure being the most common. While some tools achieved state-of-the-art performance using only sequence information [48,49,63], a few others saw an improvement in a small fraction of proteins when secondary structure information [33,44,64] was added. However, our previous work did not observe an improvement in performance when combining secondary structure with sequence information. ENNGene was the first study using evolutionary conservation to predict RBP binding sites that proved it to be an important input feature improving the model performance [31].

Following our previous findings, we further investigated the influence of various input features on predicting RBP binding sites, with the presumption of RNA sequence being the most crucial feature, and evolutionary conservation together with the secondary structure being additional features potentially improving the prediction capability of the models. We developed four model types for each combination of inputs: SCR RBP-24, SCR RBP-31, TL RBP-24, and TL RBP-31. Our results clearly indicate the importance of RNA sequence, as S models outperformed EC models in most cases, except for a slight improvement in SCR EC models on RBP-31. However, combining S and EC together improved performance even above the S models, especially when using the SCR approach. These results support the value of the EC feature in predicting RBP binding sites.

In contrast, the performance of models that only used the SS input was subpar, while combining S and SS resulted in a decline in performance across both SCR and TL models. However, TL models that utilized all three inputs showed a slightly different trend. The best results were achieved with the S + EC + SS combination, resulting in a small increase in performance on both benchmarks. Full three-branched TL models slightly surpassed other variants in 17 out of 24 proteins in RBP-24 and 26 out of 31 in RBP-31. For SCR models, the best performance was achieved using the S+EC combination, with the addition of SS resulting in a performance decrease.

It is well known that some RBPs have well-determined RNA binding domains, and thus preferences in their target RNA sequences, which they recognize in a “static” manner. Most of the proteins in both datasets fall into this category, such as PUM2, ELAVL1/HUR, HNRNPC, or IGF2BP1-3, and for those proteins, we generally observed RNA sequence as a dominant input feature when the prediction scores of the S models were compared to the models trained using the other two input features (Supplementary Tables S2 and S3). However, other RBPs do not seem to have a sequence preference and bind RNA in a dynamic manner: for instance, helicase MOV10 or eIF4AIII. For those, we would expect the RNA sequence to have lower scores than the predicted RNA secondary structure, but the obtained scores for only SS proteins remained the lowest. On the other hand, interestingly, we may see that evolutionary conservation can be as important as sequence, or sometimes even more important for such proteins. That raises a potentially interesting topic for future research in the field, as secondary structure patterns might be more visible to the model from the EC patterns than from the RNA sequence itself. Finally, the mRNA target of the miRNA-loaded Ago2 is not determined by Ago2 but by miRNA loaded on the protein in a DICER complex. It is well known that the sequence of many miRNAs is found to be conserved, which was very well visible from our results when all our EC models obtained much higher AUC scores than S models on all Ago datasets, especially in RBP-31. Despite all the interesting results, deep investigation of binding characteristics of individual proteins is beyond the scope of our study, so we at least publish obtained results for all the proteins in both datasets for those interested.

Overall, the results of our study indicate that the RNA sequence is the most important input feature for predicting RBP binding sites, with evolutionary conservation providing a significant improvement in model performance. In contrast, using predicted secondary structure alone showed subpar performance, and combining it with the sequence did not improve the results significantly. The predicted SS sequences are artificial and based solely on our current knowledge, which could introduce bias. We suggest that exploring alternative sequence encodings besides the predicted secondary structures could further improve model performance and accuracy in predicting RBP binding sites. Our results also highlight the potential for using transfer learning approaches in RBP binding site prediction, with TL models consistently outperforming SCR models, regardless of the combination of inputs.

In addition to investigating the impact of individual input features, we also explored the effect of input interval length on the performance of the TL method. We found no significant difference in AUC scores for RBP-24 when varying interval lengths from 100 nt to 300 nt. For RBP-31, although a small increase in AUC scores was observed with increasing interval length, no statistically significant difference was detected. These results suggest that the TL method is robust and can perform well with varying interval lengths. However, the optimal interval length may vary by particular RBP dataset. Future studies could explore the impact of interval lengths on other methods and specific RBP datasets. For the rest of our current study, we decided to continue with the interval length of 150 nt, which has been reported to be optimal for RNA secondary structure prediction [34].

The concept of Transfer Learning [26] was introduced as a technique to optimize training processes by addressing limitations imposed by dataset size. In our study, we evaluated the efficiency of Transfer Learning by comparing our pre-trained BS model fine-tuned on unseen data from the PTBv1 dataset, which was not included in the baseline dataset, to a model of the same architecture trained from scratch. Initially, we used the BS model to predict the PTBv1 evaluation dataset. The results were nearly random, dispelling concerns that the BS was learning biases typical of CLIP experiments rather than crucial binding features of the proteins. Following the prediction with the fine-tuned TL model and model trained from scratch, the AUC scores showed a slight increase in favor of the TL method, yielding a performance highly competitive with other published methods.

As PTBv1 is one of the largest datasets within RBP-24, we further investigated whether the TL method’s superiority is consistent across various RBPs datasets with different sample sizes. To accomplish this, we evaluated the performance of TL and SCR models on the PTBv1 dataset and four additional proteins from RBP-24 with varying dataset sizes. By subsampling these datasets to various sizes, ranging from 100 samples per class to the entire dataset, we demonstrated the remarkable performance gains of the TL method. The TL method outperformed the SCR models by almost 30% on datasets with only 100 samples per class. The gap in performance between TL and SCR models narrowed between 5000 and 10,000 samples per class, suggesting that the TL models can obtain satisfactory results even with only a few hundred samples, while SCR models require at least 10,000 samples per class to achieve comparable results.

Observing the promising performance improvements on small datasets, we conducted an overall comparison with other published methods, using all datasets of varying sizes combined in both benchmarks. Our TL method achieved an average AUC score of 0.944 on the RBP-24 dataset, which is on par with the scores obtained by other state-of-the-art methods. On the RBP-31 dataset, our TL method also performed well, with an average AUC score of 0.897, slightly outperforming the current state-of-the-art method iCircRBP-DHN [25].

Our study has shown that the TL method is a promising approach for improving the performance of models for predicting RBP target sites. RBP target site prediction is critical in understanding post-transcriptional gene expression regulation. However, there are still proteins for which obtaining large amounts of experimental data is challenging. For instance, it may be difficult to precipitate certain proteins, or they may not respond well to UV crosslinking [10,11]. Having accurate prediction models can aid in discovering new protein–RNA interactions and their roles in various biological processes. Our contribution to this field lies in demonstrating that TL can enable reliable predictions even with limited data, making it a valuable tool for proteins with only a few hundred experimentally validated genomic intervals.

The attention scores of our models also provided evidence of their ability to identify relevant RNA subsequences, with the extracted *k*-mers showing clear differences between individual proteins and being consistent with literature-derived motifs. This suggests that our models have successfully learned the specific characteristics of the RBPs, further supporting their reliability and potential usefulness for future research in this field.

## 5. Conclusions

In this study, we explored the use of transfer learning in predicting RNA-binding proteins’ target sites. To accomplish this, we designed a three-branched neural network that integrated elements used with success in previous studies in the field, such as a hybrid CNN-RNN network, an attention layer, and residual networks.

To evaluate the benefits of this approach, we applied it to two widely recognized CLIP-seq RBP data collections, RBP-24 and RBP-31, with distinct properties such as interval length, negative sample derivation method, positive-negative ratio, and individual dataset sizes. We optimized the model’s performance using a novel hyperparameter tuning pipeline that reduced tuning time by 19% on average.

Our findings demonstrate that combining RNA sequence and evolutionary conservation leads to superior model performance compared with using sequence alone. In addition, we found that transfer learning provides a significant performance boost over models trained from scratch in both data collections. Most importantly, we show that transfer learning is particularly advantageous when working with datasets with limited experimental data, as satisfactory results can be achieved with just a few hundred samples. In contrast, the commonly used approach of training models for each protein separately requires much larger datasets to produce similar results. Our findings emphasize the potential of transfer learning in future studies, particularly for proteins with limited data. Furthermore, we demonstrate how incorporating the attention layer into the network can aid the visualization and verification of predictions in a biologically relevant context.

In summary, our study demonstrates the success of transfer learning in predicting RBP binding sites, particularly in challenging scenarios with limited data or an unbalanced positive-negative ratio. We hope our findings will enable the development of more accurate models for RBP binding site prediction in the future.

## Figures and Tables

**Table 1:** Tuned parameters were the number of units in the BiGRU layer, embedding dimension, tokenizer size, number of filters in CNN layers, and a number of ResNet blocks and fully-connected layers. Individual stages are described in the text above (Section 2.6).

Stage	Hyperparameter	Search Space	Total Combinations
1	BiGRU layer units	64, 128, 256, 512	16
	Embedding dimensions	16, 32, 64, 128	
2	BPE tokenizer size	16, 32, 64	3
3	Filters in CNN layers	32, 64,128	6
	Number of ResNet blocks	2, 3	
4	Number of fully connected layers	2, 3	2

**Table 2:** Comparison of experimentally obtained processing times for our hyperparameter optimization pipeline and Random Search algorithm.

Run Number:	Time in Hours						
	1	2	3	4	5	AVG	Std
Our optimization method	13.755	12.713	13.625	14.109	13.663	13.573	0.518
Random Search (Keras)	16.173	16.095	15.665	16.035	15.961	15.986	0.196

## Data Availability

The data and code presented in this study are openly available at https://github.com/VaculikOndrej/TransferLearningRBP.

## Supplementary Materials

The following supporting information can be downloaded at: https://www.mdpi.com/article/10.3390/biology12101276/s1, Figure S1: Comparison of our method trained using different approaches on individual datasets in RBP-24; Figure S2: Comparison of our method trained using different approaches on individual datasets in RBP-31; Figure S3: Most important 6-mers obtained from trained TL model for each dataset in RBP-24; Figure S4: Most important 6-mers obtained from trained TL model for each dataset in RBP-31; Table S1: Manually extracted average AUC values from published methods that used RBP-24 as benchmark set. The table shows difference in the average performance based on the number of samples in the individual datasets; Table S2: Comparison of the individual branches model performance measured in AUC scores on RBP-24 dataset, PTBv1 was excluded as described in the paper; Table S3: Comparison of the individual branches model performance measured in AUC scores on RBP-31 dataset.
