# Supplementary material for: Transfer Learning Allows Accurate RBP Target Site Prediction with Limited Sample Sizes
Source: Biology (Basel). 2023 Sep 25;12(10):1276. doi: 10.3390/biology12101276 (PMC10604046; doi:10.3390/biology12101276)
Supplement: Supplementary file 1 [file biology-12-01276-s001.zip › TableS1.pdf]

Table S1. Manually extracted average AUC values from published methods that used RBP-24 as benchmark set. The table shows difference in the average performance based on the amount of samples in the individual datasets.

	Number of training samples			
**Method**	**<10K**	**>10K**	**All**	**Source**
**GraphProt**	0.865	0.901	0.887	[17]
**deepnet-rbp**	0.880	0.916	0.903	[33]
**DeepBind**	0.882	0.939	0.918	[20]
**iDeepV**	0.869	0.939	0.913	[60]
**iDeepE**	0.905	0.946	0.931	[61]
**DeepRKE**	0.902	0.953	0.934	[51]
**PBVPP**	0.950	0.955	0.953	[64]
**DeepMSA**	0.933	0.958	0.949	[62]
**EDCNN**	0.924	0.956	0.944	[63]
**ENNGene**	0.942	0.949	0.947	[31]
