# Supplementary material for: Transfer Learning Allows Accurate RBP Target Site Prediction with Limited Sample Sizes
Source: Biology (Basel). 2023 Sep 25;12(10):1276. doi: 10.3390/biology12101276 (PMC10604046; doi:10.3390/biology12101276)
Supplement: Supplementary file 1 [file biology-12-01276-s001.zip › TableS3.pdf]

Table S3. Comparison of the individual branches model performance measured in AUC scores on RBP-31 dataset.

	RBP-31		
**Protein**	**Seq**	**Cons**	**SecStruct**
**Ago/EIF**	0.717	**0.857**	0.574
**Ago2-MNase**	0.594	**0.695**	0.515
**Ago2-1**	0.836	**0.913**	0.534
**Ago2-2**	0.852	**0.900**	0.535
**Ago2**	0.621	**0.638**	0.476
**eIF4AIII-1**	0.932	**0.962**	0.606
**eIF4AIII-2**	**0.938**	0.935	0.553
**ELAVL1-1**	**0.920**	0.777	0.637
**ELAVL1-MNase**	0.584	**0.714**	0.535
**ELAVL1A**	0.854	**0.861**	0.678
**ELAVL1-2**	**0.933**	0.887	0.698
**EWSR1**	**0.899**	0.792	0.689
**FUS**	**0.946**	0.709	0.660
**Mut FUS**	**0.939**	0.829	0.656
**IGFBP1-3**	0.681	**0.829**	0.565
**hnRNPC-1**	**0.953**	0.672	0.622
**hnRNPC-2**	**0.978**	0.552	0.599
**hnRNPL-1**	**0.699**	0.661	0.514
**hnRNPL-2**	**0.652**	0.564	0.530
**hnRNPL-like**	0.643	**0.676**	0.519
**MOV10**	0.814	**0.827**	0.473
**Nsun2**	0.751	**0.767**	0.634
**PUM2**	**0.959**	0.878	0.599
**QKI**	**0.958**	0.703	0.721
**SRSF1**	**0.883**	0.873	0.625
**TAF15**	**0.767**	0.554	0.591
**TDP-43**	**0.909**	0.645	0.535
**TIA1**	0.867	**0.875**	0.608
**TIAL1**	**0.890**	0.774	0.602
**U2AF2**	**0.969**	0.830	0.585
**U2AF2(KD)**	**0.923**	0.805	0.550
