# Supplementary material for: Transfer Learning Allows Accurate RBP Target Site Prediction with Limited Sample Sizes
Source: Biology (Basel). 2023 Sep 25;12(10):1276. doi: 10.3390/biology12101276 (PMC10604046; doi:10.3390/biology12101276)
Supplement: Supplementary file 1 [file biology-12-01276-s001.zip › TableS2.pdf]

Table S2. Comparison of the individual branches model performance measured in AUC scores on RBP-24 dataset, PTBv1 was excluded as described in the paper.

	RBP-24		
**Protein**	**Seq**	**Cons**	**SecStruct**
**ALKBH5_Baltz2012**	0.800	**0.878**	0.578
**C17ORF85_Baltz2012**	0.869	**0.895**	0.617
**C22ORF28_Baltz2012**	0.853	**0.895**	0.581
**CAPRIN1_Baltz2012**	0.823	**0.863**	0.568
**CLIPSEQ_AGO2**	0.879	**0.888**	0.570
**CLIPSEQ_ELAVL1**	**0.976**	0.837	0.644
**CLIPSEQ_SFRS1**	**0.931**	0.849	0.628
**ICLIP_HNRNPC**	**0.975**	0.540	0.629
**ICLIP_TDP43**	**0.919**	0.596	0.605
**ICLIP_TIA1**	**0.941**	0.820	0.602
**ICLIP_TIAL1**	**0.938**	0.784	0.585
**PARCLIP_AGO1234**	**0.878**	0.867	0.563
**PARCLIP_ELAVL1**	**0.957**	0.738	0.679
**PARCLIP_ELAVL1A**	**0.976**	0.843	0.670
**PARCLIP_EWSR1**	**0.954**	0.771	0.625
**PARCLIP_FUS**	**0.979**	0.682	0.638
**PARCLIP_HUR**	**0.989**	0.711	0.701
**PARCLIP_IGF2BP123**	**0.898**	0.828	0.609
**PARCLIP_MOV10_Sievers**	0.860	**0.871**	0.594
**PARCLIP_PUM2**	**0.954**	0.842	0.635
**PARCLIP_QKI**	**0.974**	0.642	0.721
**PARCLIP_TAF15**	**0.986**	0.741	0.667
**ZC3H7B_Baltz2012**	0.786	**0.796**	0.538
