# Supplementary figures and images for: Transfer Learning Allows Accurate RBP Target Site Prediction with Limited Sample Sizes
Source: Biology (Basel). 2023 Sep 25;12(10):1276. doi: 10.3390/biology12101276 (PMC10604046; doi:10.3390/biology12101276)

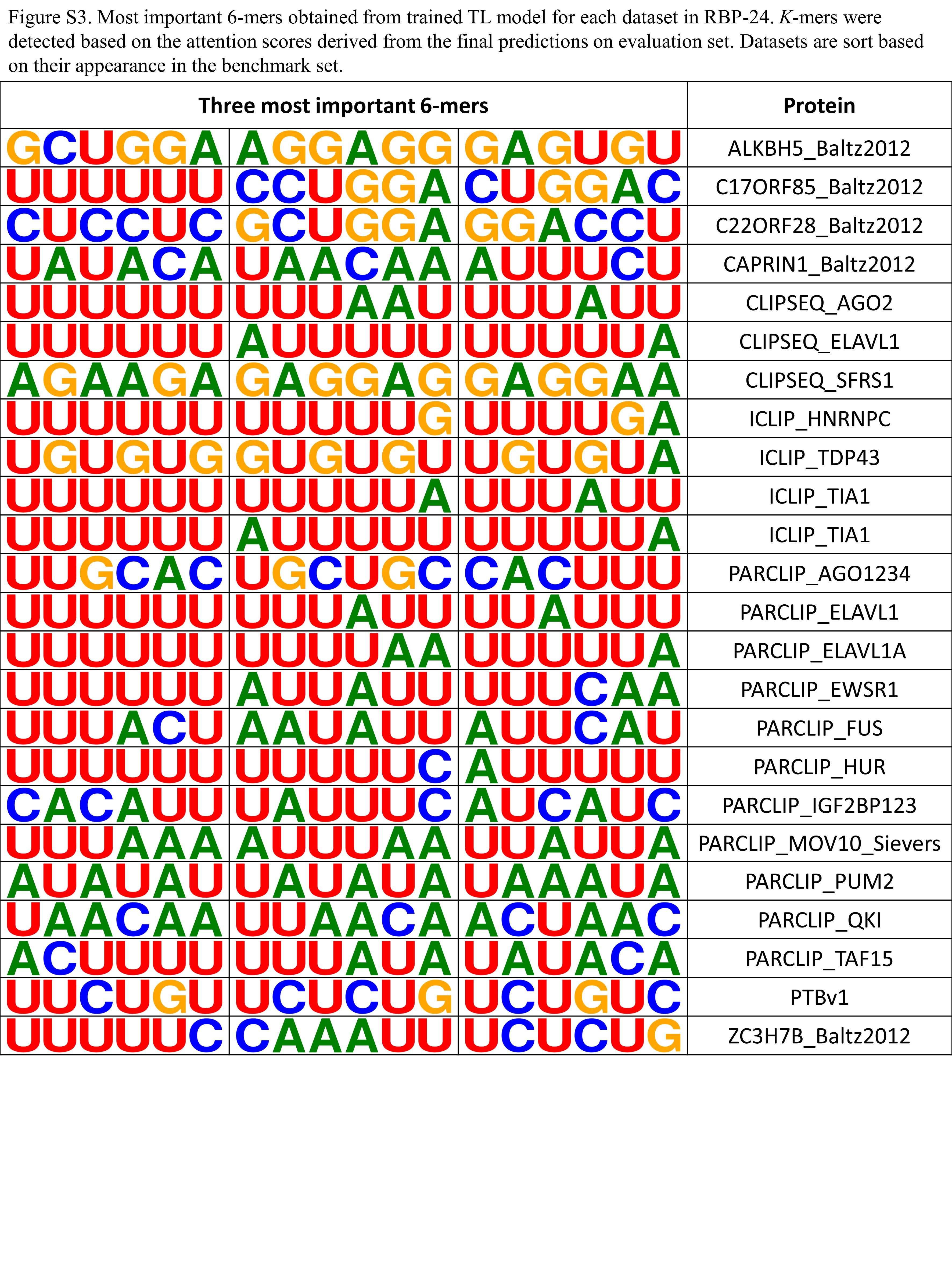

Supplement: Supplementary file 1 [file biology-12-01276-s001.zip › FigureS3_interpretationKmersRBP24.jpg]

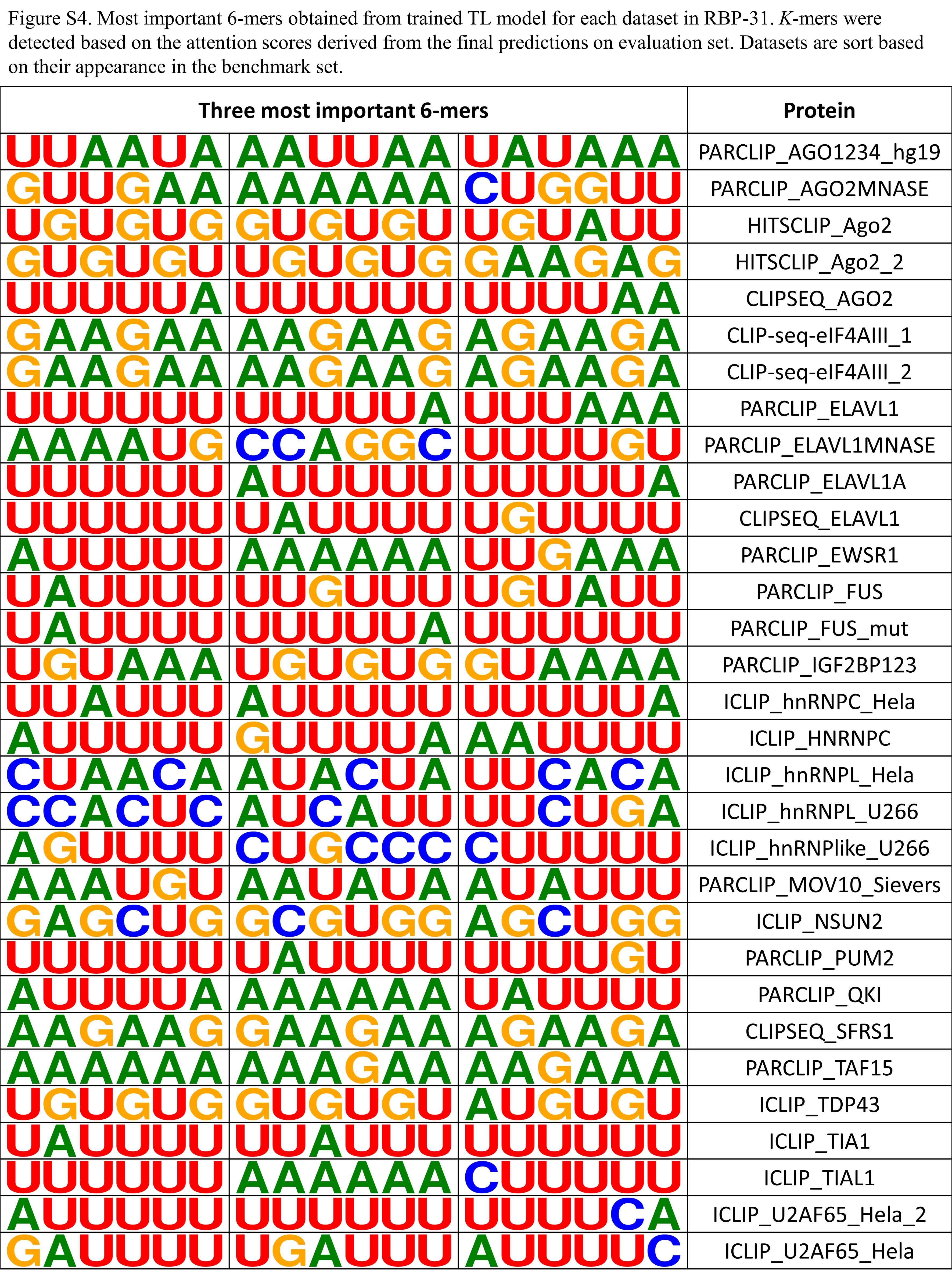

Supplement: Supplementary file 1 [file biology-12-01276-s001.zip › FigureS4_interpretationKmersRBP31.jpg]

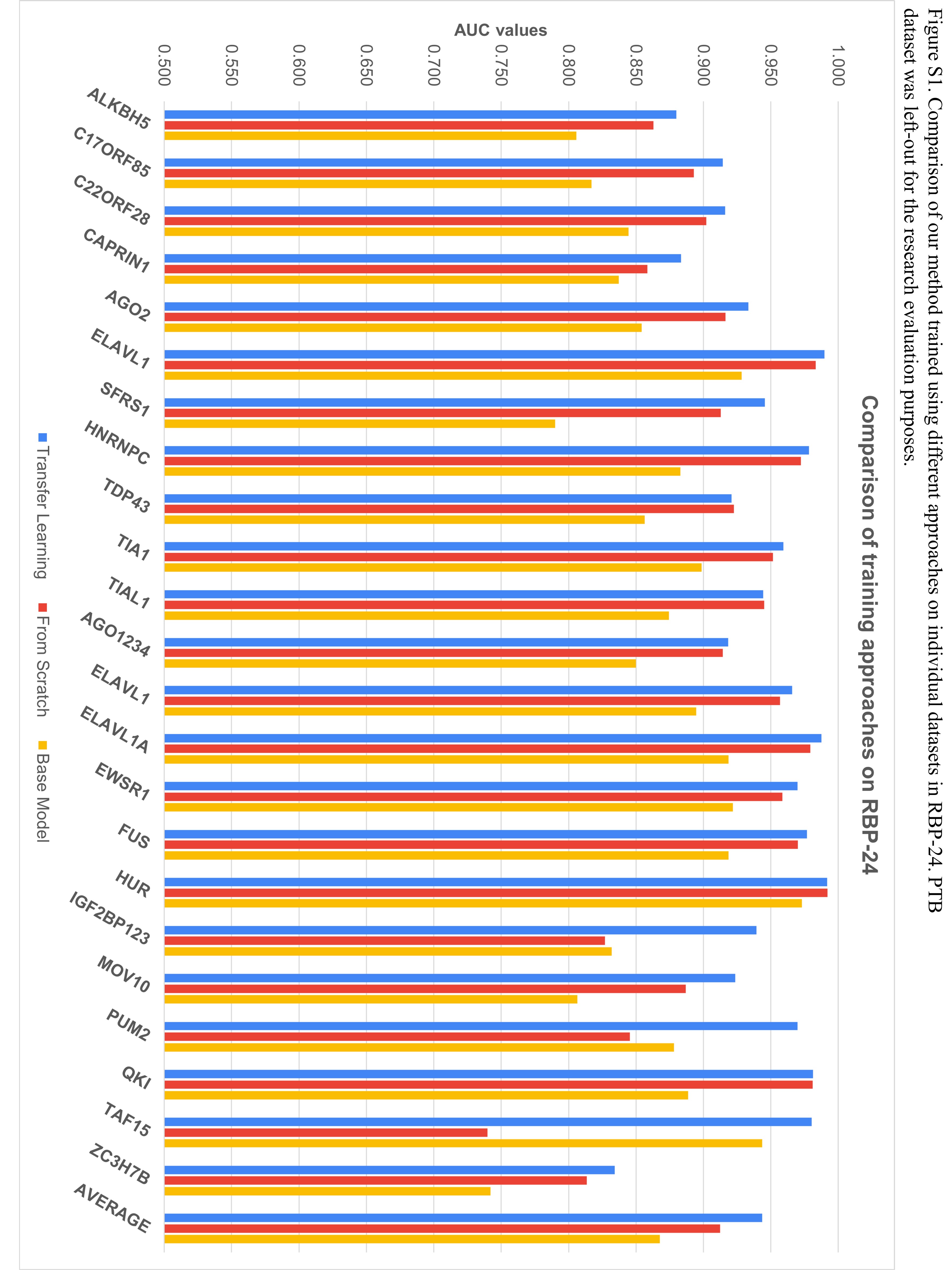

Supplement: Supplementary file 1 [file biology-12-01276-s001.zip › FigureS1_trainingApproachesRBP24Comparison.jpg]

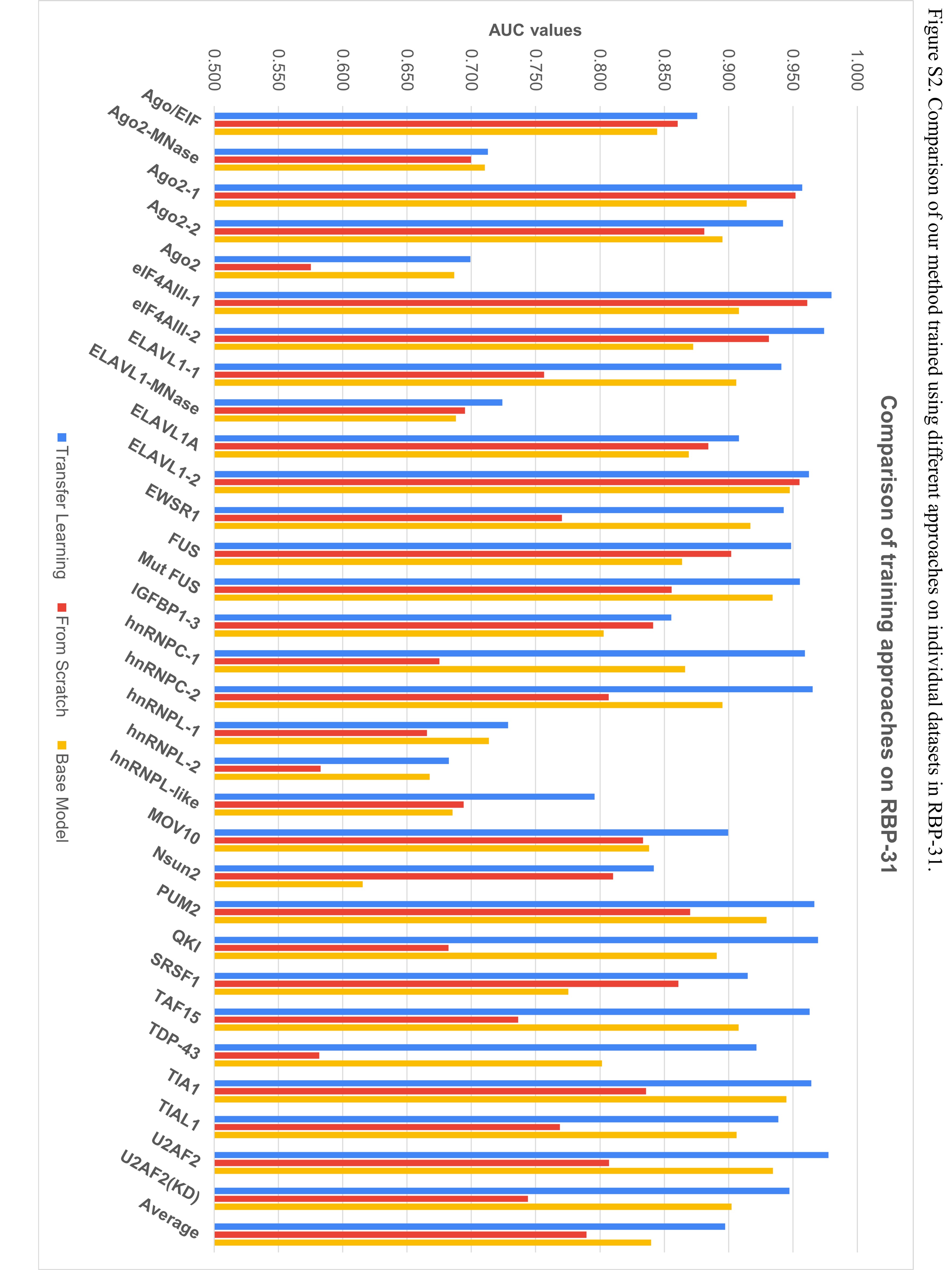

Supplement: Supplementary file 1 [file biology-12-01276-s001.zip › FigureS2_trainingApproachesRBP31Comparison.jpg]
